# Comparison of 3D and 2D acquisition of late gadolinium enhancement in patients with acute, subacute and chronic myocardial infarction

**DOI:** 10.1186/1532-429X-13-S1-P154

**Published:** 2011-02-02

**Authors:** Robert Goetti, Sebastian Kozerke, Olivio F Donati, Paul Stolzmann, Roberto Corti, Robert Manka

**Affiliations:** 1 University Hospital Zurich, Zurich, Switzerland; 2 ETH Zurich, Zurich, Switzerland

## Purpose

To assess a late gadolinium enhancement (LGE) imaging single breath-hold 3D inversion recovery sequence for the quantification of myocardial scar mass and transmurality in comparison to a clinically established 2D acquisition sequence.

## Methods

Ninety patients (84 men, age 54.4±10.8y, BMI 27.8±4.5 kg/m^2^) with acute (n=30), subacute (n=30) and chronic (n=30) myocardial infarction were included in the study. All imaging was performed on a 1.5-T clinical MR system (Achieva, Philips Medical Systems, Best, the Netherlands). Spatial resolution was identical for 3D and 2D images (1.5 x 1.5 mm^2^, slice thickness 8 mm, no slice gap). Image quality was graded on a five-point scale (1: excellent, 5: non-diagnostic). Quantitative analyses of myocardial mass (g), scar mass (g) and scar transmurality (five-point scale: 0: 0%; 1: <25%; 2: <50%; 3: <75%; 4: 75%-100%) were performed. Intra- and interobserver agreement were assessed for 15 randomly chosen patients (5 of each group).

## Results

Mean image quality was not significantly different in 3D (1.50±0.675) and 2D (1.41±0.669; p=0.26) datasets. Non-diagnostic image quality (score: 5) did not occur. Acquisition time was significantly shorter for 3D datasets (26.7±4.4 sec vs. 367.7±56.4 sec; p<0.001). There were no significant differences between 2D and 3D datasets in mean myocardial mass (2D: 148.3 ± 35.1 g; 3D: 148.1 ± 34.6 g; p=0.76) and scar tissue mass (2D: 31.8 ± 14.6 g; 3D: 31.6 ± 15.5 g; p=0.39) with strong and significant correlation between 2D and 3D datasets regarding both myocardial mass (r=0.982; p<0.001) and scar tissue mass (r=0.980; p<0.001). Bland-Altman analysis showed a mean difference of 0.21±6.64 g (range: -19.64 - 18.44 g) for myocardial mass and a mean difference of 0.26±2.88 g (range: -7.15 - 7.74 g) for scar mass between 2D and 3D datasets. Agreement between the two acquisition techniques regarding scar transmurality was excellent for the detection of non-viable segments (>50% scar tissue transmurality; κ = 0.81) and was good (κ = 0.75) for the more detailed assessment using the five-point transmurality score. Inter- and intra-observer agreements were good to excellent (κ = 0.70-0.90).

## Conclusions

3D LGE imaging enables accurate quantitative evaluation of scar tissue mass and transmurality with significantly shorter acquisition time compared to 2D LGE imaging.

**Figure 1:**
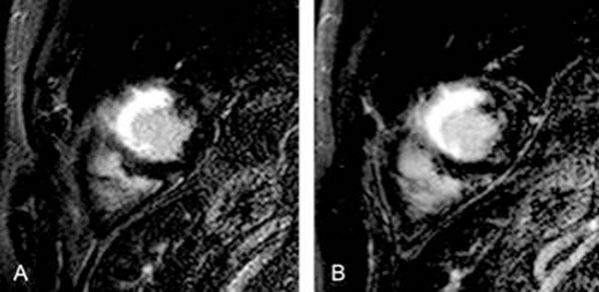
Images of 2D (A) and 3D (B) acquisitions in a 48 y/o male with acute myocardial infarction showing equal image quality and delayed enhancement extent.

